# Design and Development of a Low-Cost Optical Current Sensor

**DOI:** 10.3390/s131013584

**Published:** 2013-10-10

**Authors:** Joseba Zubia, Luciano Casado, Gotzon Aldabaldetreku, Alfonso Montero, Eneko Zubia, Gaizka Durana

**Affiliations:** 1 Department of Communications Engineering, ETSI de Bilbao, University of the Basque Country (UPV/EHU), Alameda de Urquijo s/n, Bilbao 48013, Spain; E-Mails: gotzon.aldabaldetreku@ehu.es (G.A.); gaizka.durana@ehu.es (G.D.); 2 Smart Grid, Derio Bidea 28, Zabalondo Industrialdea, Mungia 48100, Spain; E-Mails: loc@arteche.es (L.C.); AOM@arteche.es (A.M.); eoz@arteche.es (E.Z.)

**Keywords:** Faraday rotation, plastic optical fibre, optical fibre sensors, current sensor

## Abstract

In this paper we demonstrate the design of a low-cost optical current sensor. The sensor principle is the Faraday rotation of a light beam through a magneto-optical material, SF2, when a magnetic field is present. The prototype has a high sensitivity and a high linearity for currents ranging from 0 up to 800 A. The error of the optical fibre sensor is smaller than 1% for electric currents over 175 A.

## 1. Introduction

The new uses of the electrical network, as well as the increasing demand for greater quality of service (QoS), require providing the network with “intelligence”. After the technological revolutions of the last few decades, with unimaginable technological advances in the area of information and communications technology (ICT), the electrical sector had turned into an anachronism using the technology of last century. One of the necessary challenges will be to obtain information about the primary elements of the network at any point. Optical technology is an ideal candidate for this, without the safety requirements (disconnectors, lightning rods, *etc.*) of conventional sensors.

Optical current transformers based on optical fibre sensors are now being developed worldwide [1–4]. In comparison with conventional transformers, optical fibre current sensors (OFCS) are compact and lightweight, easy to install, immune to electromagnetic noise, and they offer a wide measurement range and long distance signal transmission [5–8]. To date, a number of OFCS have been shown to be feasible. They have good reliability and sensitivity, although the price is also high. This disadvantage can be overcome with plastic optical fibre (POF).

POFs are a cheap and robust counterparts to glass optical fibres. For short haul applications and sensors, POFs offer many advantages, such as high numerical aperture, huge core diameter, ease of connection and good mechanical reliability, among others [9,10]. In many sensing applications, the distance between the measuring points and the electronics, including the emitter and the receiver, is relatively short [11–14]. This feature, as well as its immunity to electric and magnetic fields, makes POFs suitable for electric current sensing [15].

The basic physics principle behind the sensor operation is the modulation of the polarization of light. The rotation of the polarization vector of light within a transparent material along the direction of an external magnetic field was first reported by Michael Faraday in 1845 [16]. An interesting aspect of the Faraday rotation is that the sense of rotation relative to the direction of the magnetic field is independent of the light direction for a given material. Most of the sensors developed are based on garnets or flint glasses with a large Verdet constant [17–19], although a magnetic field sensor made of bulk PMMA has also been proposed [15].

The goal of this work has been the design and development of a low-cost optical current sensor, including its modular electronics. The novelty of the design is two-fold: on the one hand, the optical head of the new sensor makes use only of plastic components (POF, collimators and polarizers) to transmit and receive the light from a magnetooptic glass with small excess losses. On the other hand, the novelty of the electronics relies on the combination of a low-noise and a high-sensitivity design that makes it useful for the measurement of low current values with minimal errors.

There is a wide range of applications of this sensor such as the control of electrolysis process for the production of metals, monitoring of currents on overhead electrical distribution lines, in underground electrical vaults, currents within Switchgear, monitoring magnetic fields and fault detection applications, among others.

This work has been developed in collaboration with the Smart Grid Company of the ARTECHE group, which is dedicated to the electricity sector. Recently, a pilot installation using optical fiber sensing technology was successfully commissioned at COPEL's 138 kV Posto Fiscal substation in the state of Paraná, Brazil. In this paper, the principle of operation of the optical current sensor will be explained first, including a detailed description of the optical head and the electronics. Afterwards, a characterization of the sensor will be given, together with some examples of field results with the sensor. Finally, the main conclusions will be summarized.

## 2. Theory

**Figure 1.**
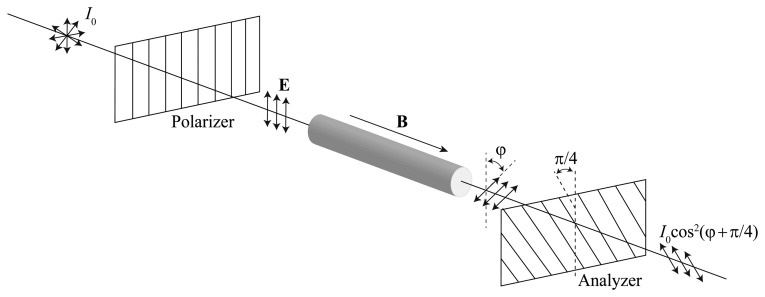
Faraday rotation.

The Faraday effect was the first magneto-optical effect to be proposed and demonstrated in fibre current sensors. Figure 1 shows a schematic configuration of the Faraday rotation. When a light beam passes through a glass medium in a magnetic field, its polarization vector is rotated in proportion to the field. The Verdet constant V relates the line integral of the magnetic field **B** to the rotation of the polarization plane of a linearly polarized, according to the equation [20–22]:

(1) φ=V⨖Bdl

Since **B** is proportional to the current, *φ* is also directly proportional to the current. Equation (1) states that the Faraday rotation and, hence, the sensitivity of the crystal, increases with the Verdet constant *V* and it also shows that the longer the optical path (crystal rod length *d*), the greater the Faraday rotation. These parameters are both important and give us two degrees of freedom to optimize the optical head of the sensor.

**Figure 2.**
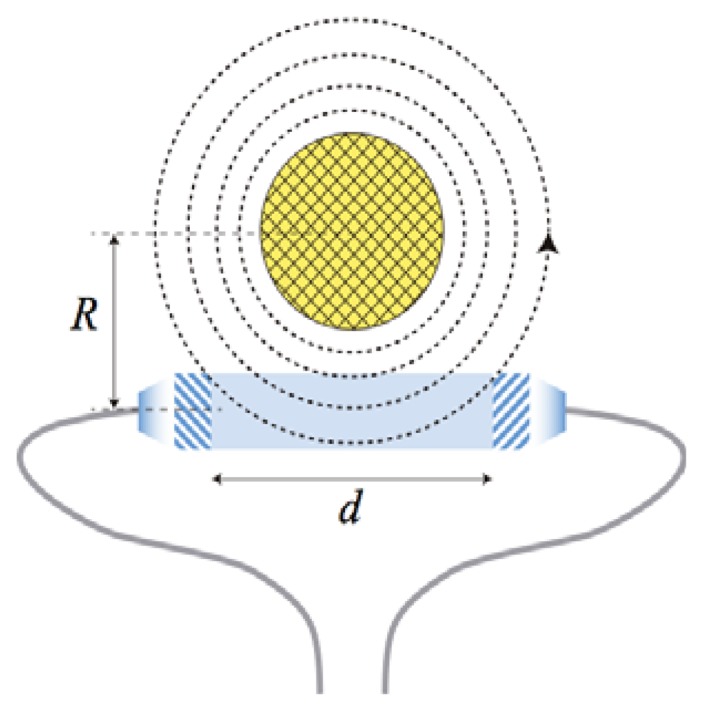
Geometry of our design showing the magnetic field generated by a metallic wire (yellow) around the SF2 glass rod (blue).

The Verdet constant has positive values for diamagnetic materials, such as the SF2 glass used in our design, and negative values for paramagnetic materials [17–19]. Figure 2 depicts the geometry of our specific approach, with the glass rod placed perpendicular to the metal wire. The magnetic field **B** produced by the current of a metallic wire is:

(2) $$B=\frac{\mu_{0}i}{2\pi R}$$

where *R* is the distance from the wire axis, *i* is the electric current and *μ*_0_ the permeability of free space [23]. If the length of the SF2 glass rod *d* is small enough, the magnetic field can be considered constant inside the glass material and Equation (1) reads:

(3) $$\varphi \approx VBd=\frac{\mu_{0}Vid}{2\pi R}$$

which is the basic equation governing the design of the optical head in our approach.

## 3. Design of the Current Sensor

Basically the low-cost current sensor design consists of two parts: the optical head and the accompanying electronics. This section describes both parts individually.

### 3.1. Optical Head

**Figure 3.**
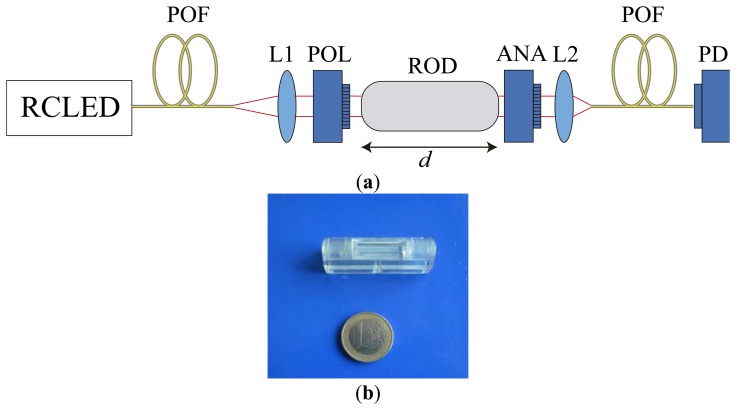
(**a**) Schematic representation of the optical head of the low-cost current sensor. L1, L2: collimating lenses; POL: polarizer; ROD: magnetooptical rod; ANA: analyser; PD: optical photodetector. (**b**) Close-up view of the magnetooptical rod.

The optical part is represented in Figure 3. It consists of two POF pieces of 5–10 m length, two PMMA collimators with a small focal length, two linear polarizer-sheets, the magneto-optic glass rod, the emitter and the receiver. The magneto-optic rod (sensing material) is a Flint glass SF2 from Schott with a Verdet constant of 11.6 rad/Tm at 632.8 nm, a diameter of 5 mm and a length of 20 mm. The collimators are biconvex plastic lenses with a focal length of 5.4 mm and an overall diameter of 7.8 mm. We also use an analog fibre optic transceiver, the FC300T from Firecomms, with termination for bare POF. It combines a high speed RCLED-based 650 nm emitter and a high sensitivity PIN diode for detection [24].

Light is transmitted from the light source to the sensor optical head via a short section of POF. The POFs used are polymethylmethacrylate (PMMA) fibres with a core diameter of 980 μm and a numerical aperture of 0.5. The unpolarized light from the RCLED passes through the collimator. The collimator transforms the divergent light into a parallel beam before reaching the linear polarizer-sheet. We use a collimator to reduce excess losses along the light path. When the light passes through the SF2 rod, the plane of polarization rotates according to the intensity of the magnetic field. After passing through the analyser, another collimator focuses light into another section of POF, which transmits the modulated intensity of light to the detector. One of the advantages of this set-up is that the optical part can be placed close enough to the current conductor without disturbing the distribution of the magnetic field and, therefore, the accuracy of the current measurement.

In the basic scheme of the polarimetric readout (Figure 1), the principal axes of the polarizer and the analyser are oriented at 45° with respect to each other in order to obtain a good linearity of the sensor response. Thus, the output irradiance is:

(4) $$I=I_{0}\cos^{2}(\frac{\pi }{4}+\varphi )$$

where *φ* is given by Equation (3). Then:

(5) $$I=I_{0}\cos^{2}\left(\frac{\pi }{4}+Vid\frac{\mu_{0}}{2\pi R}\right)=I_{0}\cos^{2}\left(\frac{\pi }{4}+2\times 10^{-7}\frac{Vid}{R}\right)$$

By making use of trigonometric equalities:

(6) $$I=\frac{I_{0}}{2}\left[1-\sin\left(4\times 10^{-7}\frac{Vid}{R}\right)\right]$$

For *d*/*R* ∼ 1, and *V*= 11.6 rad/Tm (SF2 at 632.8 nm):

(7) $$\begin{array}{l} I=\frac{I_{0}}{2}\left\{1-\sin\left[4.64\times 10^{-6}i\left(t\right)\right]\right\}=I_{AC}+I_{DC};\\ \left\{ \begin{array}{l} I_{AC}=\frac{-I_{0}}{2}\sin\left[4.64\times 10^{-6}i\left(t\right)\right]\\ I_{DC}=\frac{I_{0}}{2} \end{array}\right. \end{array}$$

which means that the AC component of the irradiance *I*_AC_ ranges from 0 to *I*_0_/2 when the electric current goes from 0 to around 340,000 A for the SF2 glass. When the electric current goes from 0 to 800 A, we will obtain a minimum power loss of at least 11.59 dB (at 800 A), which sets the maximum output power.

The output photodiode current of the receiver, which is directly proportional to the optical power reaching the PIN photodiode, is equal to:

(8) $$\begin{array}{cc} \begin{array}{c} i_{\text{photodiode}}=\frac{I_{0}ℜ}{2}\left\{1-\sin\left[4.64\times 10^{-6}i\left(t\right)\right]\right\}\\ =i_{\text{photodiode}}^{\text{AC}}+i_{\text{photodiode}}^{\text{DC}}; \end{array} & \{\begin{array}{c} i_{\text{photodiode}}^{\text{AC}}=\frac{-I_{0}ℜ}{2}\sin\left[4.64\times 10^{-6}i\left(t\right)\right]\\ i_{\text{photodiode}}^{\text{DC}}=\frac{I_{0}ℜ}{2} \end{array} \end{array}$$

where ℜ is the responsibity of the transceiver (ℜ ∼ 0.3 for the FC300T transceiver;. Therefore, by measuring the photodiode current, the electric current through a power transmission line can be found. The uncertainties related to variations of the optical path can easily be eliminated by normalizing the AC output current $i_{\text{photodiode}}^{\text{AC}}$ with the DC output current $i_{\text{photodiode}}^{\text{DC}}$. This way, the sensor response depends neither on the light power nor on the misalignment of the optical components.

### 3.2. Electronics

**Figure 4.**
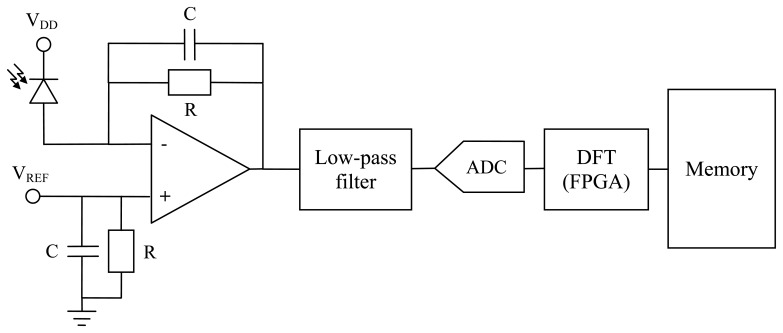
Electronic set-up for the acquisition of the optical current sensor signals.

Figure 4 shows the electronic set-up for the acquisition of the current sensor response signals. The photodiode converts the optical signal to an electric current. This electric signal consists of two components (refering back to Equation (8)): an alternating current (AC) component, due to the effect of magnetic field in the rod, and a direct current (DC) component, due to the light rectification. Both components are amplified using a low input bias current operational amplifier. The gain of the amplifier is equal to the value of the resistance R. R must be of a very high value (≫10 k& ohm;) to accommodate the electric signal to the analog to digital converter (ADC) when the current through the power transmission line is the maximum (*i.e.*, 800 A). The V_REF_ voltage is used to compensate de DC component of the light in order to accommodate the electric signal to the ADC.

The amplified signal is then filtered to avoid aliasing effects. Afterwards, the signal is sampled using a fast 16 bits ADC. The samples are processed using a digital signal processing algorithm on a FPGA device, which calculates for each cycle the 50 Hz (AC component) and 0 Hz (DC component) discrete Fourier transform (DFT) of the current signal [25]. The DFT values are stored in a memory which is accessible from a laptop.

**Figure 5.**
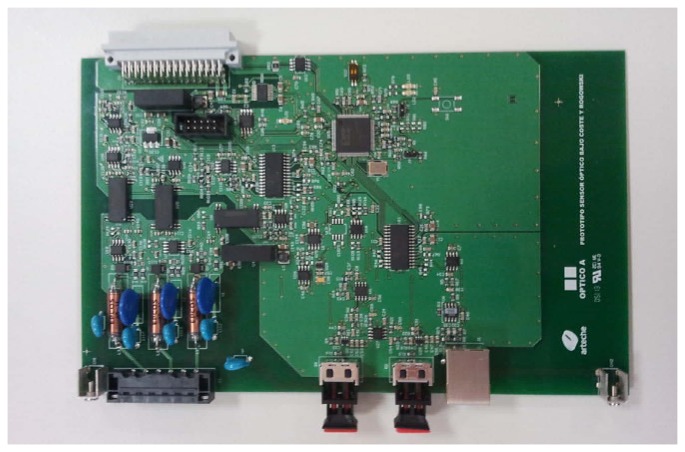
Custom electronic hardware for the acquisition of the current sensor signals.

Figure 5 shows the custom hardware developed for the acquisition of the low-cost optical current sensor signals (the hardware includes the Firecomms FC300T fibre optic transceiver). Due to the very weak optical signals received for low currents across the conductor, signal integrity in the hardware design has been of very high importance [26].

The custom electronic hardware is modular and it can easily be used in a rack together with other existing sensors (resistive sensors, toroidal sensors, *etc.*) that measure other parameters of the electric transmission lines.

## 4. Experimental Section

### 4.1. Characterization of the Optical Head

Optical excess losses in the optical head depend on both the quality of the components and the geometry of the design. We have calculated these excess losses taking into account emitter-POF coupling losses, POF transmission losses, Fresnel reflections at any air-plastic or *vice versa* interfaces, collimators and polarizers absorption losses, and, finally, POF-receiver coupling losses. We have also included losses due to the misalignment of the optical components (POF-collimator-rod), which is critical to couple the light into the SF2 rod and to couple it back into the receiving fibre. We have compared this calculus with a direct measurement of the excess losses of the optical head. To accomplish this task we have measured the optical power across the optical head and through a piece of POF. Excess losses are just the light power difference. By adding all these contributions we have estimated a theoretical value of 20 dB, whereas the experimental measurements resulted in a value of 16 dB. The small disagreement is due to the conservative character of the theoretical calculus of the sources of loss, since we have assumed the worst-case scenario for the mechanical tolerances.

### 4.2. Results

**Figure 6.**
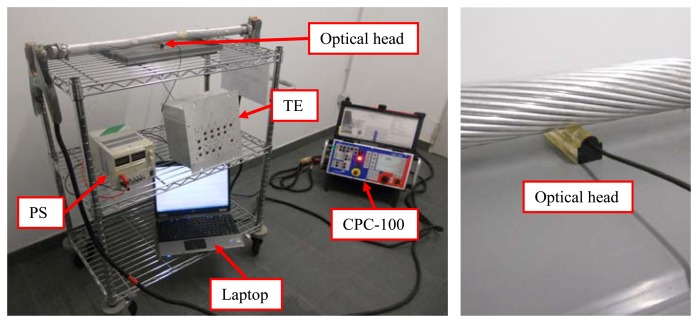
Test set-up of the low-cost optical current sensor (**Left**). Optical head detail (**Right**). TE: test equipment, including the electronic hardware for the acquisition inside. PS: power supply. Laptop: used to acquire all the data. Optical head: optical head of the current sensor.

Laboratory tests were carried out using a conductor with a diameter of 5 cm. Figure 6 shows the experimental set-up of the low-cost optical current sensor and the electronics described in previous sections. The optical head of the current sensor was attached to the conductor using a dual-side adhesive tape. Different current values were injected to the conductor using a CPC-100 universal testing device by Omicron [27]. The CPC-100 can generate current values ranging from 0 A to 800 A. The testing device was connected in series to the conductor using means provided by the manufacturer. Care was taken to place the sensor far enough from the CPC-100 to reduce the 50 Hz interference from the testing device.

**Figure 7.**
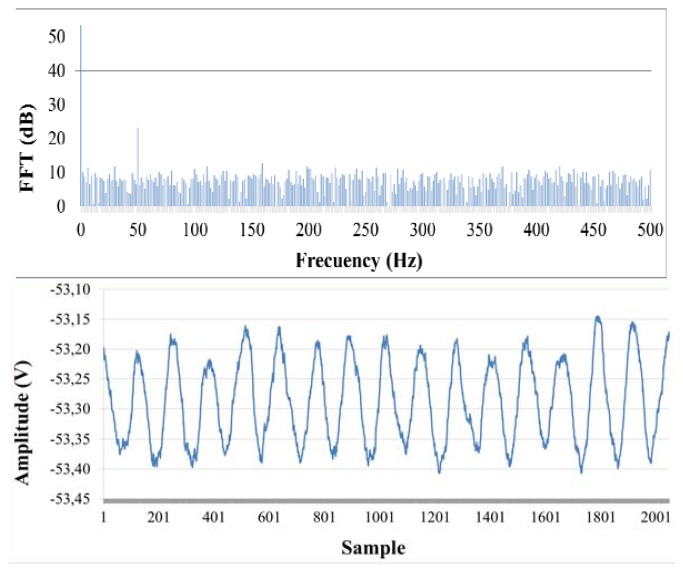
Optical signal received after amplification for *I* = 400 A (**Above**). DFT of the optical signal from 0 to 500 Hz (**Below**).

The low-cost optical current sensor was tested for different current values, ranging from 0 A up to 800 A. For each current value, 256 samples of DFT were stored in memory and the arithmetic mean was calculated. The DFT results were normalized to the DC component values, in order to cancel uncertainties due to variations in the optical path or light fluctuations. For instance, Figure 7 shows the representation of the signal received for *I* = 400.

**Figure 8.**
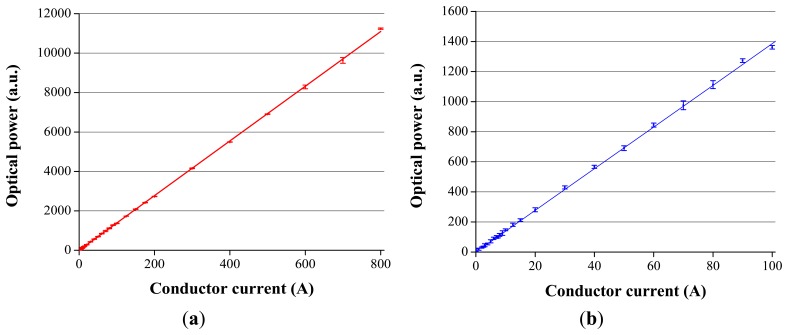
(**a**) 50 Hz component of the normalized DFT from *I* = 0 A to *I* = 800 A. (**b**) 50 Hz component of the normalized DFT from *I* = 0 A to *I* = 100 A.

We have plotted the 50 Hz component of the DFT as a function of the current in Figure 8. In both cases the measurements were repeated five times, and the mean value and its least mean squared curve-fit was calculated. It is clear from the mean value (red squared curve in the first case and blue squared curve in the second case) that the response of the current sensor exhibits a high degree of current linearity, a fact assessed by the *R*^2^ coefficient derived from the fitting process (*R*^2^ = 0.99986). The best fit is represented by a red line in Figure 8a and by a blue line in Figure 8b. The vertical error bars denote the uncertainties associated to each current measurement.

For current values lower than 20 A, the response of the sensor is affected by the noise of the received optical signal and the floor noise of the acquisition system.

For current values greater than 30 A, the measurement uncertainty is less than 5%. In order to measure with an error lower than 1%, the current must be greater than 175 A. This improves the results obtained by other authors with glasses composed of oxides of heavy metals as well as telluric glass [28] or optical fibers doped with CdSe quantum dots [29], whose errors, in the same current range, are around 20%. However, they are worse than other optical current sensors based on single mode fibers in an interferometric configuration. Accuracy and repeatability of these sensors are within ±0.1% over a wide range of current and temperatures [30,31]. Their counterpart is their much higher price and great complexity.

On the other hand a toroidal current transformer with ferromagnetic core, conventionally used in field applications for current measurement, has an error of less than 1% for current values greater than 40 A. Therefore, the accuracy of the low-cost current sensor is slightly lower than that of conventional current sensors. Nevertheless, the accuracy of our current sensor could be improved in future prototypes by separating the DC and AC components before analog to digital conversion or by improving the optical head of the current sensor using better magneto-optical rods (increasing the price goal). The mechanical characteristics of the optical head could also be improved to reduce signal loss due to optical misalignment. All in all, we believe that our current sensor prototype is accurate enough for applications in which the precision at low current values is not critical, such as fault detect applications.

## 5. Conclusions

A new low-cost optical current sensor has been developed. The sensing principle, its optical and electronic design, as well as its characterization have been described. The performance of the prototype was tested experimentally. Our current sensor is low cost, with good reliability, and has a linear response from 0 to 800 A with an accuracy over 1% for current values over 175 A. Additionally, the modular electronics developed for the sensor can easily be fitted in a rack together with other existing sensors measuring other parameters of the electric transmission lines. Although there is a wide range of applications for this sensor, it will mainly used to monitor currents both on overhead and underground electrical distribution lines.
